# Interaction of soil pH and phosphorus efficacy: Long-term effects of P fertilizer and lime applications on wheat, barley, and sugar beet

**DOI:** 10.1007/s13280-017-0970-2

**Published:** 2017-11-24

**Authors:** Sabine von Tucher, Dorothea Hörndl, Urs Schmidhalter

**Affiliations:** Technical University of Munich, Emil-Ramann-Straße 2, 85354 Freising, Germany

**Keywords:** CAL-extractable P, Long-term P experiment, P fertilizer recommendation, P use efficiency

## Abstract

Phosphorus (P), a plant macronutrient, must be adequately supplied for crop growth. In Germany, many soils are high in plant-available P; specifically in arable farming, P fertilizer application has been reduced or even omitted in the last decade. Therefore, it is important to understand how long these soils can support sustainable crop production, and what concentrations of soil P are required for it. We analyzed a 36-year long-term field experiment regarding the effects of different P application and liming rates on plant growth and soil P concentrations with a crop rotation of sugar beet, wheat, and barley. Sugar beet reacted to low soil P and low soil pH levels more sensitively than wheat, which was not significantly affected by the long-term omitted P application. All three crop species showed adequate growth at soil P levels lower than the currently recommended levels, if low soil pH was optimized by liming. The increase in efficacy of soil and fertilizer P by reduced P application rates therefore requires the adaptation of the soil pH to a soil type-specific optimal level.

## Introduction

Phosphorus (P) is one of the main plant nutrients, and its deficiency substantially reduces plant growth. Therefore, farmers in Germany were advised to establish P reserves in low-P soils by applying amounts of P that greatly exceeded the plant P demand and removal. The basic concept of this recommendation was to sustain the current biomass production by these soil P reserves that were thought to be optimal for plant growth. The relationship between the soil reserves of plant-available P and biomass productivity was established by the definition of “nutrient availability classes” based on the topsoil tests of P soluble in calcium acetate/calcium lactate (CAL) or double-lactate (DL) (Kerschberger et al. 1997; Kuchenbuch and Buczko 2011). When soils had reached their “optimal” P level, it was recommended to maintain these levels by re-application of the amount of P lost by its removal from harvested plant products. However, the soil P levels for optimal plant growth are a subject of permanent debate (Köster and Schachtschabel 1983; Taube et al. 2015). Recent results of these discussions (summarized in Taube et al. 2015) therefore suggest to reduce the recommended optimal range for lactate-soluble soil P from now 4.5 to 9.0 mg P 100 g soil^−1^ to 3.0 to 6.0 mg P 100 g soil^−1^ in the future.

Nevertheless, the up to now recommended strategy caused highly positive P input–output balances up to the 1980s. At this time, plant production did not focus on the negative consequences of P losses on surface water quality. Although the average P input–output balances were significantly reduced from the 1990s, most soils in Germany are sufficiently or even highly supplied with plant-available P (Tóth et al. 2014; Claassen et al. 2015). The latter is specifically true for the regions with intensive livestock husbandry. This “legacy P” poses high and long-term risks to water bodies (Sharpley et al. 2013).

However, negative P balances were established specifically in the arable farms without animal husbandry during the last decades. In the last 10 years, the import and consumption of mineral P fertilizer in Germany remained more or less constant at a level where the P input to the agricultural land was lower than the P removal by the harvest products (Eurostat 2016). In soils where P application was decreased or even omitted in the last decades, the P availability might have become too low for an optimal crop production. For such situations, it still remains to be determined how long these soil P reserves allow for adequate crop yields and what concentrations of soil P are required for optimal crop production. In addition, to avoid further environmental risks by P losses, it is crucial to answer whether low-P soils should be stocked up with new P reserves, or whether sufficient crop production is possible at soil P levels relatively lower than the currently recommended levels.

In German soils, most of the P is adsorbed to aluminum or iron oxides and hydroxides, bound as calcium-phosphates, or present in organic forms (Sims and Pierzynski 2005). The soil pH plays an important role in influencing the plant availability of P. Therefore, besides the application of adequate P amounts, the application of lime needs to be considered for optimizing the soil pH.

Under P shortage, plant species employ a number of different adaptation strategies to overcome or alleviate its deficiency (Raghothama 1999; Hinsinger 2001; Ho et al. 2005). These mechanisms include morphological adaptations, e.g., changes in root-length densities or symbiosis with mycorrhizal fungi as well as chemical mobilization of the sparingly soluble P by root exudates with an aim of increasing P acquisition. The efficiency of these adaptation strategies differs among various plant species.

Due to the high phosphorus buffering capacity of soils, the effect of different management practices on P availability can be most convincingly assessed after their long-term application. This is particularly true when the effect of omitted P fertilizer application on the reserves of plant-available P over time is to be studied. Depending on the soil P level, the soil type, and the soil organic matter content, significant changes in the concentration of plant-available P, for example, determined as Olsen-P, were observed only after about a decade or more (Johnston et al. 2016). In addition, plots without P application need to be compared to fertilized treatments at experimental site conditions that are comparable. If P fertilizer application should follow good agricultural practice, only moderate P rates should be applied, that do not exceed the plant P removal by more than a factor of about two. Therefore, long-term field experiments are very valuable tools to answer these questions.

In this study, we examined the long-term effects of the omitted and varied P fertilizer application with and without adequate liming on the growth and potentially different P acquisition of three field crop species: sugar beet (*Beta vulgaris* L. subsp. *vulgaris*), wheat (*Triticum aestivum* L.), and barley (*Hordeum vulgare* L.). Firstly, the long-term course of the yield and P removal of the three crops over time were evaluated and the long-term changes in plant-available P in the soil were addressed. Secondly, the P response within the last recent rotation of these crops was studied according to the long-term established different P and liming conditions in the soil.

## Materials and Methods

### Site characteristics and experimental design

The field experiment was established in 1978. The experimental site was located in the Bavarian tertiary hills of southern Germany (460 m above sea level; 0.077 rad slope; 0.81 rad aspect; 790 mm average annual precipitation; 8.4 °C average temperature). The silty loam contains 1.1% C_org_ and 0.12% N_t_. At the start of the experiment, the plant-available P in soil, determined as calcium acetate/calcium lactate (CAL)-soluble P (see below) was 5.2 mg P 100 g^−1^.

Soil pH (determined in CaCl_2_; see below) currently differs according to the three liming treatments ranging from pH 4.7–5.3 (no liming) to pH 6.0–6.4 (target level 1) and pH 6.6–6.8 (target level 2). In order to reach these two target levels, soil pH was determined regularly and lime was applied at different rates as required with 5 to 25 dt CaO ha^−1^ every 2–4 years in form of calcium oxide (since 1989) or before in form of calcium carbonate or calcium silicate. Altogether, from 1978 to 2014, 161 or 237 dt CaO had been applied to reach or keep the targets level 1 or 2, respectively. Phosphorus application treatments included a control receiving no P, and an annual P application of 22 or 44 kg P ha^−1^ as water-soluble P fertilizer (super- or triplesuperphosphate in 2014). All treatments were stationary and performed in four replicates. Other nutrients, such as nitrogen (N), potassium (K), magnesium (Mg), and sulfur (S), were applied at levels adequate for plant growth.

The crop rotation included sugar (or forage) beet, winter (or summer) wheat, and winter (or summer) barley. In this study, we focused on sugar or forage beet and winter cereals. Up to 2005, the soil tillage techniques primarily included ploughing. However, since 2006, ploughless techniques have been applied. The size of the experimental plots was 8 m length × 4 m width.

### Chemical analyses

For determination of soil pH, 20 g of air-dried, 2-mm sieved soil was mixed with 50 ml of 0.01 mol L^−1^ CaCl_2_ solution, followed by gentle stirring. The pH was measured electrometrically after 2 h, when the soil was allowed to settle. The concentration of plant-available P was determined in topsoils (0–20 or 0–25 cm) after extraction with calcium acetate/calcium lactate at pH 4.1 according to the method described by Schüller (1969). The P concentration was measured colorimetrically according to the method of Murphy and Riley (1962).

For analyzing the total P content, the harvested plant material was dried at 100 °C and ground to 0.7 mm. Subsequently, the wet digestion of the ground plant material was done with HNO_3_/HClO_4_ in open vessels up to 1992, and from 1993, in closed vessels with HNO_3_/H_2_O_2_ in a microwave oven under controlled pressure (8–10 bar) (Mars 5, CEM, Kamp-Lintfort, Germany). Phosphorus concentrations in the digested solutions were determined colorimetrically using vanadate/molybdate, and since 1998, by inductively coupled plasma optical emission spectroscopy (ICP-OES; Liberty RL, Varian, Mulgrave, Australia) at a wavelength of 213.618 nm.

### Statistical analysis

The evaluation of the long-term effects (1978–2014) on the yield of the main harvest product (grain or beet dry matter) and their respective P removal was done on the basis of the relative values with the aim of eliminating the annual weather or cultivar effects in different years. For this purpose, eight “best practice” plots, i.e., P-fertilized and limed plots, were selected in each year, and equated as 100%. The treatments to be tested, without P and with or without lime application, were expressed as relative values compared to the “best practice” treatment. For the statistical analysis of the long-term effects on crop yield, the crop P removal and CAL-soluble soil P linear regressions and correlation coefficients were calculated, followed by analysis of variance (ANOVA) and F-test using the SPSS software (IBM Statistics, version 22). For direct comparison of long-term crop differences *t* test 95% confidence intervals (CI) were calculated.

## Results

### Long-term evaluation from 1978 to 2014

**Fig. 1:**
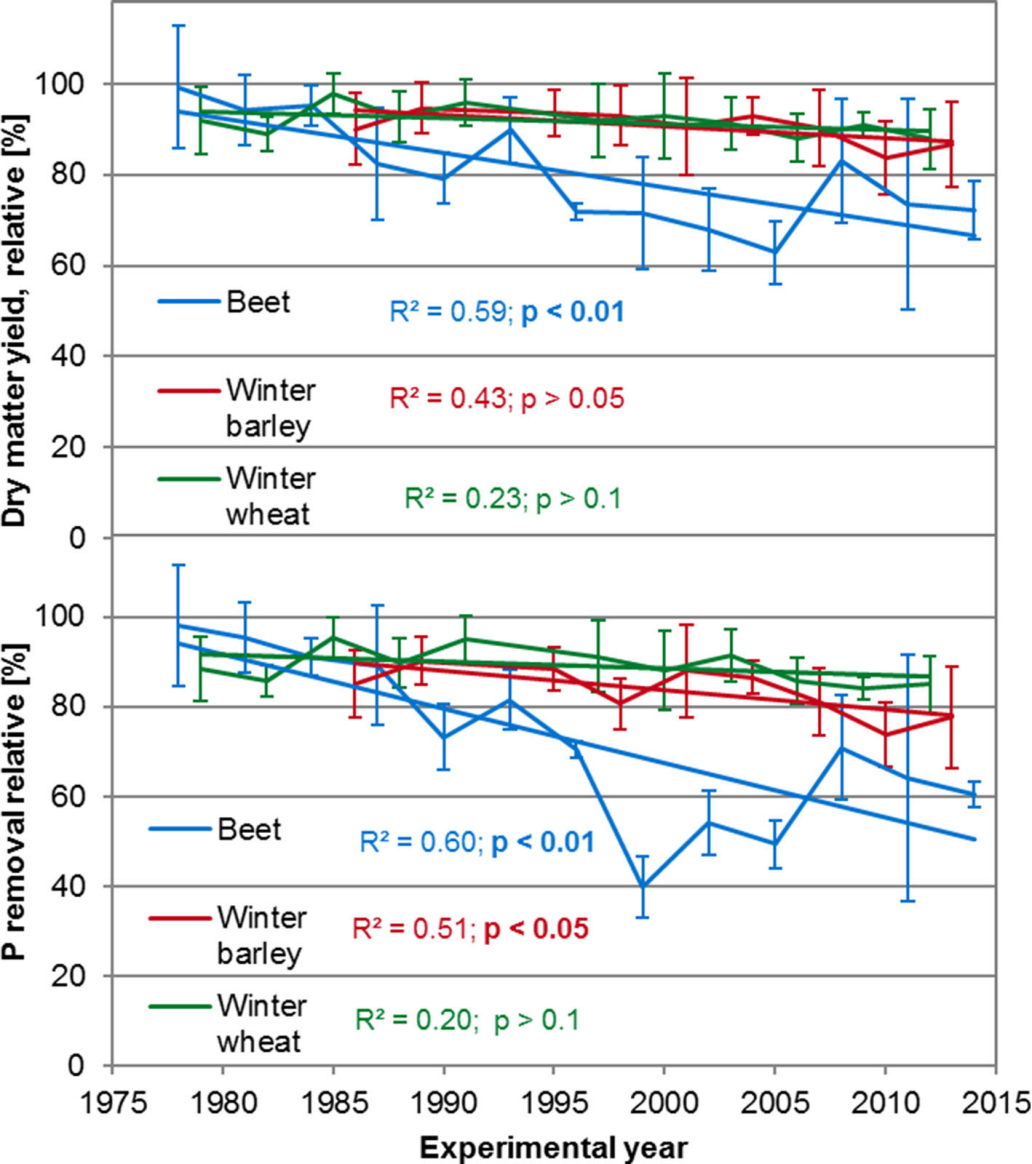
Long-term course of dry matter yield and phosphorus (P) removal of crops in plots without P fertilizer application as related to plots with optimal P supply (=100%) at soil pH 6.0–6.4

Data for the yield and P removal from 13 years of sugar or forage beet, 11 years of winter wheat, and 9 years of winter barley were available for the evaluation (Fig. 1). The long-term effects of the omitted P application on the dry matter yield of the three crops differed clearly. At the adjusted soil pH of 6.0–6.4 which had proven to be sufficient for optimal crop growth, the yield of sugar or forage beet was significantly reduced by 27% and the yield of winter barley tended to decline by 6.8%, but the yield of winter wheat remained unaffected by omitted P application. In consequence, over a period of the recent nine crop rotation cycles (Table 1) the yield reduction of sugar or forage beet (95% confidence interval (CI) 70.1–78.7% relative yield response) significantly differed from both those of winter barley (CI 88.0–93.1) and winter wheat (CI 90.0–94.3%). The analysis of P removal (Fig. 1) showed that compared to winter wheat with a reduction in P removal by 5.0%, winter barley reacted more sensitively to the long-term omitted P application because of the significant reduction in its P removal over time by 11.4%. The significant decrease in the P removal of sugar or forage beet over time by 44% from 1978 to 2014 was in agreement with the long-term P effect on the yield and confirmed the much higher sensitivity of beet crops compared with cereals (also see Table 1; 95% CIs for relative P removal in the recent nine crop rotation cycles were 56.9–67.9% for sugar or forage beet, 87.4–91.9% for winter wheat, and 80.7–86.4% for winter barley).

**Table 1:** Long-term differences in the yield and phosphorus (P) removal of sugar or forage beet, winter wheat, and winter barley as a response to omitted P application with (+) (target pH 6.0–6.4) and without (−) liming (pH 4.7–5.3). Mean values and 95% confidence intervals (CI) of relative yield and relative P removal of the recent nine crop rotation cycles are indicated. P-fertilized and limed plots were equated as 100%

	Liming	Relative dry matter yield		Relative P removal	
	±	Mean value	CI	Mean value	CI
Sugar/forage beet	+	74.4	70.1–78.7	62.4	56.9–67.9
	–	45.7	40.9–50.5	31.9	27.3–36.5
Winter wheat	+	92.2	90.1–94.3	89.6	87.4–91.9
	–	86.1	83.2–89.1	80.2	77.3–83.0
Winter barley	+	90.6	88.0–93.1	83.6	80.7–86.4
	–	82.1	77.8–86.4	72.2	67.5–76.9

If the soil pH was not increased by liming, but was allowed to remain at its site-typical low level of 4.7–5.3, the long-term effects of the omitted P application on the yield and P removal differed from those achieved by liming (Table 2). At the suboptimal soil pH, both the yield and P removal of winter barley significantly decreased over time without P application from 1986 to 2013 by 20% (slope = −0.72) and by 28% (slope = −1.03), respectively. However, for beet, a significant reduction in P removal by 46% (1978–2014; slope = −1.27), similar to the limed treatments, was observed at low pH, whereas the rate of yield reduction over time (*r* = −0.51) of beet was not statistically significant, although the relative yield of beet decreased by 26% from 64 to 38%. This is ascribed to the high variability within the replicates as well as to the fact that the low soil pH already reduced the yield of sugar or forage beet in the early years of the experiment, so that a further decrease in yield did not reach the significance level. In contrast to winter barley, the yield and P removal of winter wheat proved to be insensitive to the omitted P application over the years, and showed no significant decrease even at the low soil pH. Direct comparison of the crops within the recent 9 crop rotation cycles confirmed these crop-specific differences for yield (95% CI 40.9–50.5 for beet; 83.2–89.0 for wheat; 77.8–86.4 for barley) and for P removal (95% CI 27.3–36.5 for beet; 77.3–83.0 for wheat; 67.5–76.9 for barley) with a high sensitivity of sugar or forage beet, a moderate for winter barley and a low for winter wheat (Table 1).

**Table 2:** Long-term changes in the yield and phosphorus (P) removal of sugar or forage beet, winter wheat, and winter barley as a response to both omitted P and omitted lime application (soil pH 4.7–5.3). Equations and coefficients (*r*) of correlations with statistical significance (*F* test) are indicated

	Relative dry matter yield			Relative P removal		
		*r*	*p* value		*r*	*p* value
Sugar/forage beet	*y* = −0.71*x* + 1467	−0.51	>0.05	*y* = −1.27*x* + 2580	−0.78	<0.01
Winter wheat	*y* = −0.14*x* + 366	−0.19	>0.5	*y* = −0.34*x* + 768	−0.45	>0.1
Winter barley	*y* = −0.72*x* + 1530	−0.69	<0.05	*y* = −1.03*x* + 2123	−0.78	<0.05

In the equations “*x*” represents the experimental year

Results given in Table 1 also allow for comparing the effects of omitted liming on the crop species. Without P application dry matter yield of sugar or forage beet was reduced to 74% when lime was applied but to 46% without liming. A similar effect was observed for the P removal with a reduction to 62% with liming and to 32% without liming. The cereals, however, were much less affected by omitted liming. Their dry matter yield under omitted P application decreased to 91 (for wheat) or 92% (for barley) when limed and to 82 or 86% without liming. Effects of liming on the P removal of the cereals were somewhat higher than those on the yield.

**Fig. 2:**
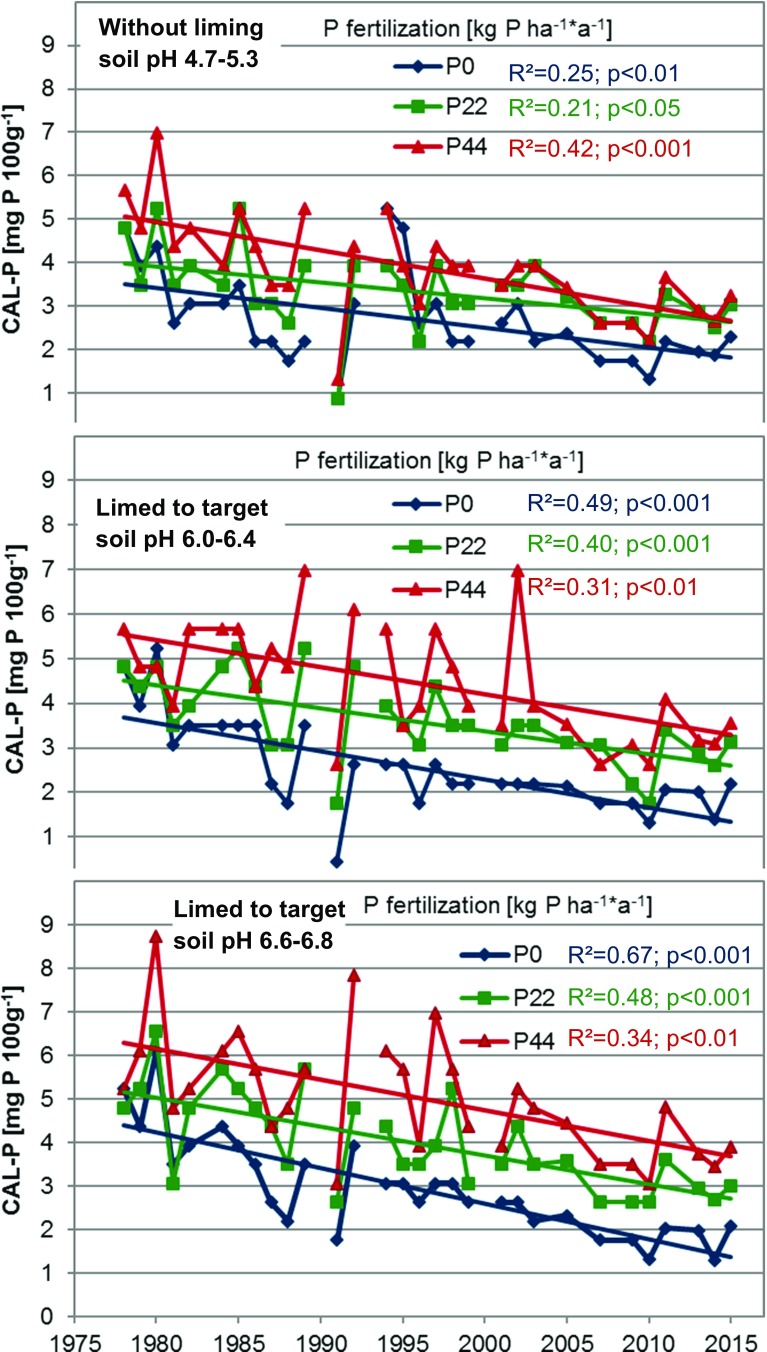
Long-term time course estimation of calcium acetate/calcium lactate (CAL)-soluble phosphorus (P) contents in topsoils with different P fertilizer application rates in limed (target pH 6.0–6.4 and 6.6–6.8) and non-limed (pH 4.7–5.3) plots

Throughout the whole experimental period, the plant-available P was determined in the topsoil (Fig. 2). Without P fertilizer application, the CAL-soluble P decreased at both low and high soil pH values, which can be explained by the annual P removal of the crops. It has to be stated that a decline was also observed when P was applied at levels of 22 and 44 kg P ha^−1^; however, these amounts of applied P were expected to meet the assessed average P removal or even exceed it. In addition, the response of CAL-soluble P extraction to P fertilizer application was affected by the soil pH value. If soil pH was low (4.7–5.3) due to omitted liming, the difference in CAL-soluble P between unfertilized and P-fertilized plots (e.g., with 44 kg P ha^−1^), particularly in the recent years of the experiment, was relatively lower compared to the limed plots with pH values of 6.0–6.4 or 6.6–6.8.

### Evaluation of one recent crop rotation cycle from 2012 to 2014

**Fig. 3:**
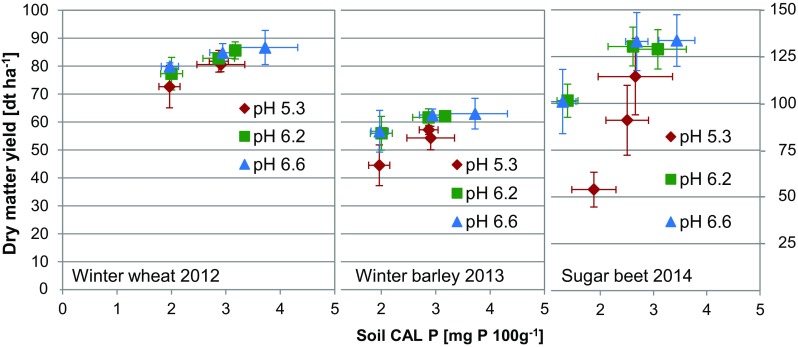
Dry matter yield of winter wheat, winter barley, and sugar beet from a recent crop rotation cycle as related to the soil calcium acetate/calcium lactate (CAL)-soluble phosphorus (P) content after long-term application of different rates of P fertilizer and liming. Error bars represent standard deviations (*n* = 4)

**Fig. 4:**
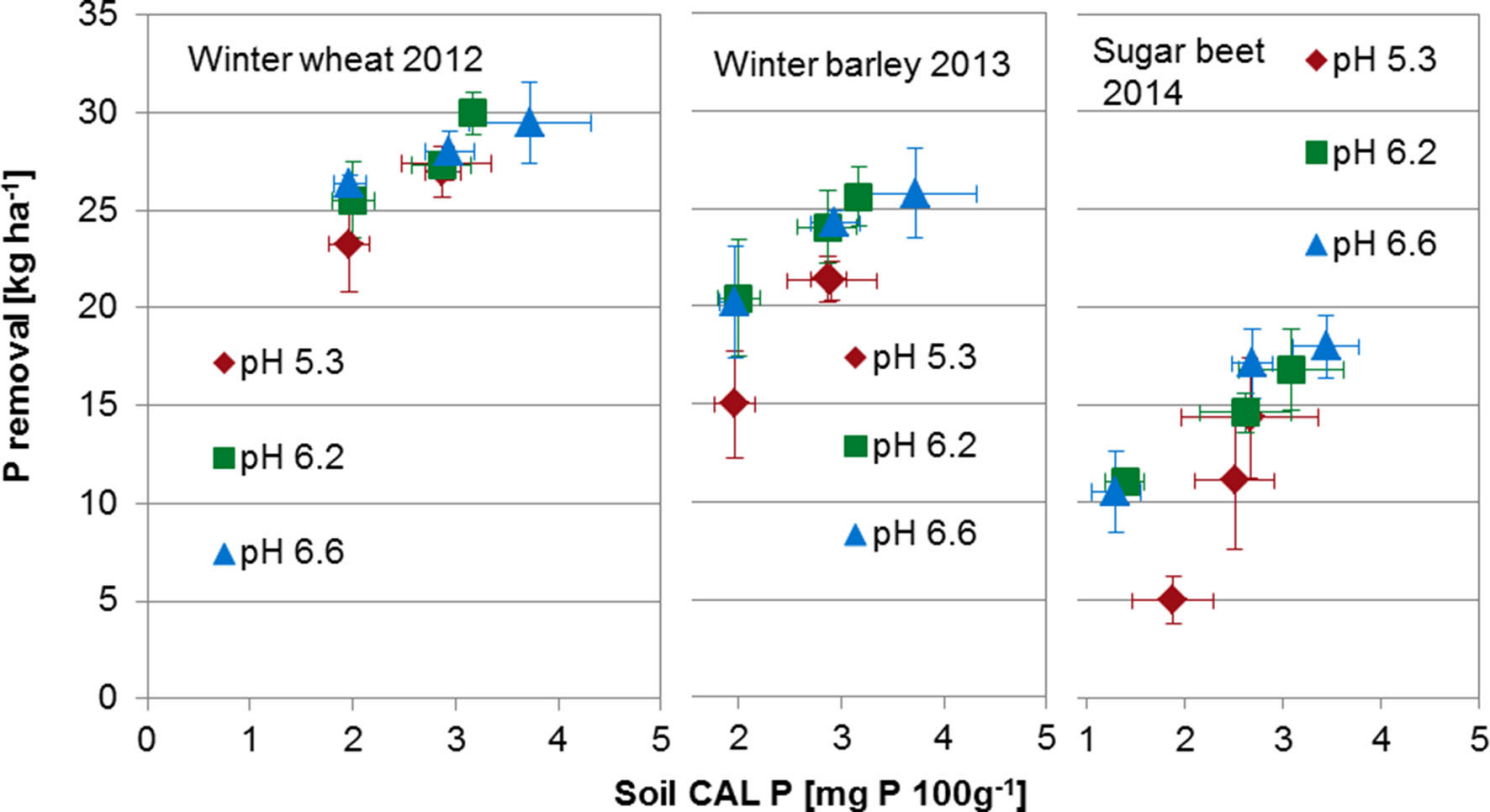
Phosphorus (P) removal of winter wheat, winter barley, and sugar beet from a recent crop rotation cycle as related to the soil calcium acetate/calcium lactate (CAL)-soluble P content after long-term application of different rates of P fertilizer and liming. Error bars represent standard deviations (*n* = 4)

The soil CAL-extractable P was related to the yield of winter wheat, winter barley, and sugar beet (Fig. 3). The most prominent reaction to both soil pH value and low lactate-soluble P was observed for sugar beet. The low levels of CAL-soluble P led to a decrease in the dry matter yield of sugar beet, but the reduction was much more pronounced when the soil pH was low, and only 47% of the yield compared to optimal conditions was achieved. In addition, at low soil pH, even intensive P fertilizer application (i.e., 44 kg P ha^−1^ year^−1^) did not result in an optimal yield response of sugar beet. In principle, similar effects of CAL-soluble P and liming were observed for winter barley and winter wheat. However, in agreement with the results from the long-term evaluation, the cereals showed relatively smaller effects on their yields at low levels of soil CAL-soluble P. A more clear difference between winter barley and winter wheat was detected when the P removal was determined (Fig. 4). The decrease in P removal by both omitted P application and liming was more pronounced for winter barley than winter wheat. However, the relationship between the soil P test with CAL and the yield of the three crops clearly states that CAL-soluble P contents of 3–4 mg P 100 g soil^−1^ were sufficient to achieve relatively high yields when the soil pH was adjusted to a soil type-specific optimized level of approximately 6.2.

## Discussion

The three investigated crop species showed different tolerance to low soil P levels. Compared to sugar beet, wheat demonstrated a long-term stability in its yield and P removal during the 36 years of omitted P application. The short-term observation of the most recent year confirmed this high tolerance to low soil P levels. Winter wheat was, therefore, more efficient to mobilize even lower soil P reserves. The relatively lower sensitivity of winter wheat compared with the beet crop is in agreement with earlier findings (Jungk et al. 1993). It might be ascribed to the differences in root properties of both species. In an oxisol with a soil pH similar to the soil used in this study, wheat showed a relatively higher root length and a higher root-to-shoot ratio compared to sugar beet (Bhadoria et al. 2002). A typical increase in the root-to-shoot ratio of wheat as a response to low P supply was also observed by Föhse et al. (1988). A high root-length density increases the spatial accessibility of the strongly adsorbed P. A similar effect of increased spatial accessibility of P is ascribed to symbiosis with mycorrhizal fungi. It was found that the wheat plants inoculated with mycorrhiza showed an increased shoot and root growth as well as a relatively higher plant P concentration compared to the wheat plants without mycorrhization (Pandey et al. 2005). The ability for mycorrhiza colonization might therefore be advantageous for the wheat plants at low soil P levels compared to sugar beet in which no mycorrhization is found.

The long-term applications of different P fertilizers led to changes in the topsoil CAL-extractable P that is used for soil testing to describe the plant P availability. When the soil pH value was adjusted to the site-specific and soil type-specific optimized levels of 6.0 or higher, an increase in P fertilizer application of 22 or 44 kg P ha^−1^ year^−1^ was reflected in CAL-soluble P. However, at low soil pH, particularly in the recent years, the difference between the two fertilizer application rates disappeared. It is, therefore, concluded that at a low soil pH, fertilizer P is more strongly adsorbed and relatively less plant-available than at a higher soil pH. This is in agreement with the growth reactions of the P-sensitive sugar beet, but is also evident for wheat and barley. Even the high P application rate of 44 kg P ha^−1^ year^−1^ could not provide enough plant-available P for optimal growth and P uptake at low pH. The interaction between the soil pH and plant P availability is in agreement with other findings, where meta-studies demonstrated that a low soil pH was a crucial factor for yield response to P application, specifically at low soil P levels (Kuchenbuch and Buczko 2011; Buczko et al. 2017). This clearly demonstrated that a too low soil pH has to be adjusted by liming to increase the use efficiency of fertilizer P. However, it can also be concluded that if the soil pH was increased to a soil type-specific optimal level, 3–4 mg CAL-soluble P 100 g soil^−1^ was sufficient to allow for adequate yields of all three tested crop species, when P fertilizer was applied annually. These findings strongly support the new strategies devised by the Association of German Agricultural Analytic and Research Institutes to reduce the soil test P availability classes by about 30% from the current range of 4.5–9.0 mg to future 3.0–6.0 mg CAL-soluble P 100 g soil^−1^ for optimal crop growth (Taube et al. 2015).

During the 36 years of experimentation, the soil CAL-soluble P decreased at the zero P treatments. Within the zero-P plots, this decrease can be explained by the annual crop P removal. However, the trend to lower the CAL-soluble P contents over time was also observed for the P-fertilized plots. The overall average crop P removal during the 36 years exactly met the first fertilizer level of 22 kg P ha^−1^ year^−1^. Therefore, the annual P input and output was exactly balanced, and the CAL-soluble P should have remained at a constant level. At a fertilizer rate of 44 kg P h^−1^ year^−1^, the average P input was twice as high as the P output, and the CAL-soluble P should have increased rather than decreased with time. The reasons for this observation are still unclear. However, recent observations indicate that the plant-available P might have moved from the investigated topsoil (0–25 cm) to the relatively deeper soil layers. The transport of phosphorus through the soil profile is thought to be associated with a bypass of the soil matrix by the preferential flow through macropores from desiccation cracks, earthworm burrows, or root channels. Preferential flow might be enhanced by reduced tillage. In comparison with the tilled systems, the no-tilled systems showed an increase in P loss loads (Djodjic et al. 2002) and led to relatively higher concentrations of dissolved P in drainage water (Williams et al. 2016). Therefore, the reduced soil tillage systems without ploughing that have been applied since 2006 might be associated with the decreasing CAL-soluble P in the topsoil of the experiment presented here. These hints of the intensified P transport to the deeper soil layers and the question whether dislocated P might significantly contribute to plant P supply will be further investigated in detail.

## Conclusions

With an aim to reduce the environmental impact by P losses from agricultural land, new strategies for P fertilizer application are needed. This study adds strong evidence to the recent discussion on the reduction of the target values for CAL-soluble soil P by about 30% as a basis for future fertilizer recommendations. The study also supports that new strategies should more consider different susceptibilities of plant species to low soil P levels as well as site-specific characteristics of P availability, in particular the soil pH values for optimized chemical and spatial P availability.

For a further refinement of P fertilizer recommendations, both additionally available P sources whether being inorganic or organic and the role of subsoil P (found below the typically analyzed 0–20 cm soil depth) in plant availability deserve closer attention in future studies under field conditions.
